# “*They cannot afford to feed their children and the advice is to stay home*. *How*‥?”: A qualitative study of community experiences of COVID-19 response efforts across Syria

**DOI:** 10.1371/journal.pone.0277215

**Published:** 2022-11-04

**Authors:** Mervat Alhaffar, Hala Mkhallalati, Omar Alrashid Alhiraki, Manar Marzouk, Ahmad Khanshour, Yazan Douedari, Natasha Howard

**Affiliations:** 1 Syria Research Group (SyRG), Co-hosted by the London School of Hygiene and Tropical Medicine and Saw Swee Hock School of Public Health, London, United Kingdom; 2 Department of Global Health and Development, London School of Hygiene and Tropical Medicine, London, United Kingdom; 3 Saw Swee Hock School of Public Health, National University of Singapore and National University Health System, Singapore, Singapore; 4 Free Aleppo University, Aleppo, Syria; Fiji National University, FIJI

## Abstract

**Introduction:**

COVID-19 highlighted the importance of meaningful engagement between communities and health authorities. This is particularly challenging in conflict-affected countries such as Syria, where social protection and food security needs can hinder adherence to non-pharmaceutical interventions (NPIs) and vaccine uptake. This study explored community perspectives of COVID-19 and health authority responses across the three main areas of control in Syria, i.e. Syrian government-controlled areas (GCA), autonomous administration-controlled areas (AACA), and opposition-controlled areas (OCA).

**Methods:**

We conducted a qualitative study, interviewing 22 purposively-sampled Syrians accessing health services in AACA, GCA, or OCA in 2021 to provide approximately equal representation by governance area and gender. We analysed data thematically using deductive and inductive coding.

**Findings:**

Interviewees in all areas described how their fears of COVID-19 and willingness to adhere to NPIs decreased as their local COVID-19 epidemics progressed and NPIs disrupted access to household essentials such as work and food. Community-level responses were minimal and ad hoc, so most people focused on personal or household protective efforts and many mentioned relying on their faith for comfort. Misinformation and vaccine hesitancy were common in all areas, linked to lack of transparency from and mistrust of local health authorities and information sources.

**Conclusions:**

The COVID-19 pandemic has increased health actors’ need to engage with communities to control disease spread, yet most NPIs implemented in Syria were inappropriate and adherence decreased as the pandemic progressed. This was exemplified by lockdowns and requirements to self-isolate, despite precarious reliance on daily wages, no subsidies for lost income, individual self-reliance, and mistrust/weak communication between communities and health authorities. We found minimal community engagement efforts, consisting entirely of informing with no efforts to consult, involve, collaborate, or empower. This contributed to failures of health actors to contextualise interventions in ways that respected community understandings and needs.

## Introduction

The COVID-19 pandemic has demonstrated the importance of controlling community disease spread through vaccination and non-pharmaceutical interventions (NPIs), e.g. safe-distancing, self-isolation, and face-masks [1]. Response interventions require significant public health awareness and willingness to engage, entailing sufficient trust and respectful relationships between health leadership, COVID-19 response organisations, and the public who are requested to adhere to mitigation measures that may be challenging or unpleasant. Transparent and respectful risk communication and health information sharing with communities is needed to address needs and concerns in adapting suitable interventions. Community engagement should ideally enable production of tailored and inclusive COVID-19 responses [2]. This is particularly important in low-income and conflict-affected settings, as precarious household income, food, and shelter can be jeopardised by insufficiently-considered COVID-19 interventions [3] or lack of trust in authorities [4, 5].

**Fig 1:**
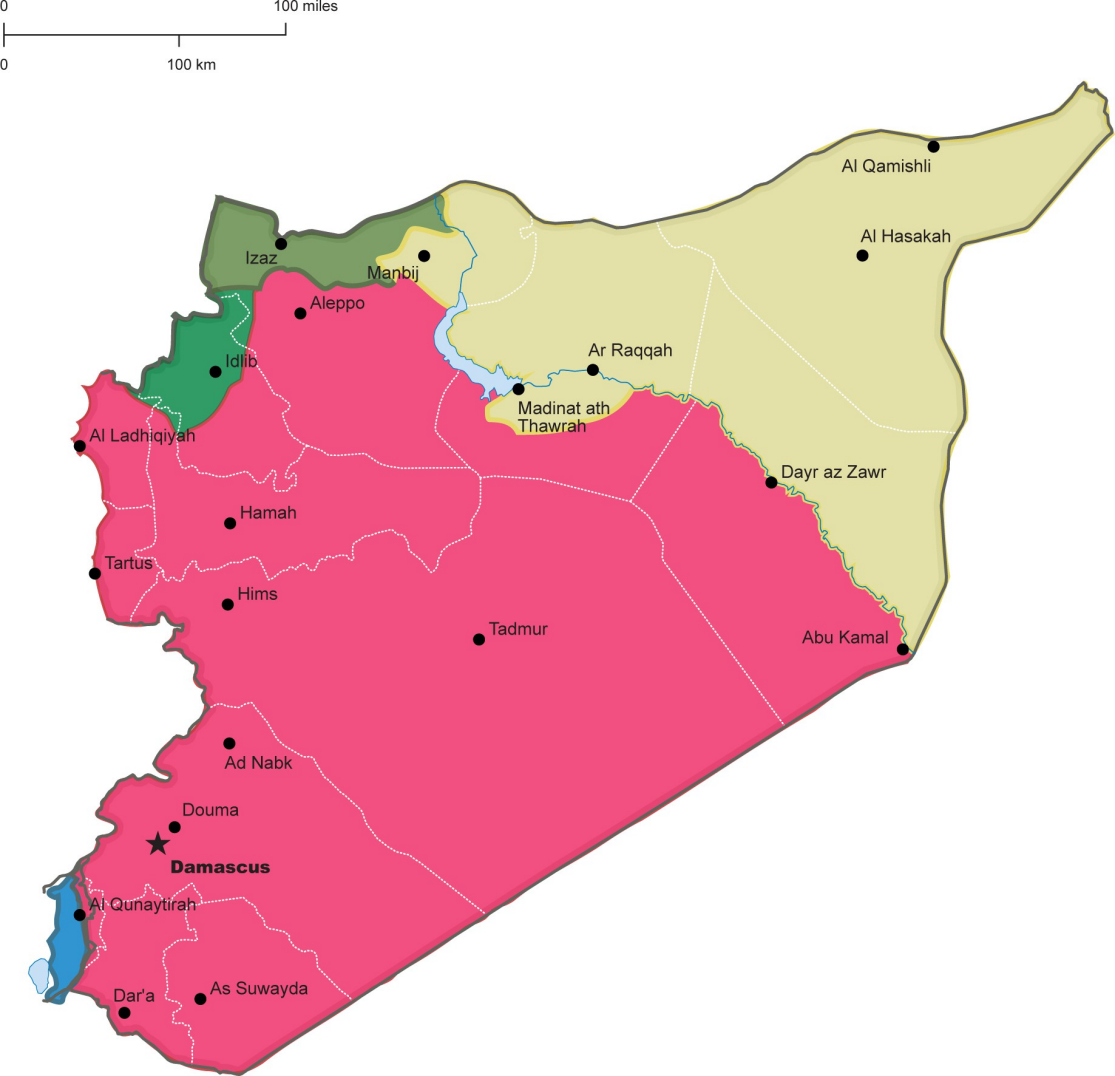
Syria map, indicating main areas of military control. NB: AACA is in yellow; GCA is in red; OCA, along with Turkish-controlled areas, are in green. Source: Noor Albeik, 2022. Additionally, an open access regularly-updated map can be found at https://syria.liveuamap.com/.

In Syria, a peaceful popular uprising in 2011 met with violent government responses and morphed into a complex, multisided conflict that has severely damaged the country, which is now categorised by the World Bank as a low-income economy [4, 6, 7]. Over 90% of the population was estimated to live under the poverty line in 2021 [8], due to economic fragility influenced by corruption, protracted conflict, sanctions, currency inflation, and deficiencies of primary commodities [9, 10]. Conflict has fragmented Syria’s health system across approximately three main territories, each with its own healthcare approaches and services, including COVID-19 responses [11]. We used the terminology of government-controlled area (GCA), opposition-controlled areas in northwest Syria (OCA), and Autonomous Administration-controlled areas in North and East Syria (AACA) for ease of reference, though each area is socio-politically heterogeneous with various actors providing health services (Fig 1). Differences in COVID-19 responses within and across these areas-of-control have affected public perceptions of the pandemic and compliance with prevention measures [10].

- GCA refers to over half the country controlled by the Syrian government, with its health system managed by the Ministry of Health in Damascus [4]. GCA’s health system lacks basic resources [12, 13] and its COVID-19 response approach has been ad hoc, ranging from initial denial to implementing partial securitised lockdowns in some areas [4, 13, 14].
- OCA refers to the shrinking geographical area controlled by the Syrian opposition, bordered by GCA, Turkiye, and AACA. OCA healthcare is organised under two independent health systems, one managed by the Idlib Health Directorate and the other from Turkiye [4, 11]. COVID-19 responses in OCA were relatively transparent, but insufficient resources, poor coordination, and large numbers of internally-displaced people—many in makeshift camps—constrained response efforts [15, 16].
- AACA refers to the large northeastern area bordered by Iraqi Kurdistan, OCA, GCA, and Turkiye, primarily controlled by the Kurdish-led Autonomous Administration [11]. Its health system is predominantly dependent on cross-border humanitarian support [17, 18]. External support was significantly disrupted by closure of Al-Yaroubiyah/Tel Kocher crossing in July 2020, with weak health facilities unable to adequately address needs [19].

The impact of the COVID-19 pandemic in conflict-affected countries manifests differently than in lower-income countries unaffected by conflict, as governance is particularly fragmented, health systems have limited capacity, and insecurity and violence threaten response efforts. In Syria, COVID-19 research is relatively limited, especially related to the experiences and practices of ordinary Syrians that can play a significant role in mitigating transmission in areas with minimal governance or local authority support [20–23]. The COVID-19 pandemic has exacerbated ongoing socioeconomic challenges [14, 24]. The first COVID-19 case was officially reported in Damascus in 22 March 2020 [14], followed by initial reported cases on 2 April 2020 in AACA [25] and 9 July 2020 in OCA [26]. Health authorities and international partners in each area implemented standard NPIs, including border and school closures, facemasks, handwashing, self-isolation, and quarantining in community isolation centres [4]. NPI coverage and uptake varied across areas-of-control but was characterised by lack of adherence [13]. COVID-19 vaccination rollout began in May 2021 with a first COVAX donation of 203,000 doses to Damascus and 53,800 doses to OCA, with additional COVAX and bilateral donations totalling approximately 4.8 million since [27]. As with NPIs, hesitancy and resistance were noted [28], without root causes being fully explored. Thus, research was needed on how COVID-19 responses could be improved across Syria.

We aimed to examine public perceptions about the effects of the COVID-19 pandemic and of health authority responses in Syria’s three main areas-of-control. Objectives were to: (i) explore lay perspectives on COVID-19 effects and health authority responses in each area-of-control; (ii) identify individual, household, or community-level prevention and mitigation efforts; (iii) and consider potential ways to improve responses at the local (i.e. individual, household, community) level. We also discuss implications for policymakers and practitioners.

## Methods

### Study design

We adopted an exploratory qualitative study design, featuring remote semi-structured interviews with adult men and women using health services in GCA, OCA, or AACA and inductive thematic analysis drawing on concepts from critical phenomenology as described in Weiss *et al* [29], to foreground experiences of marginalisation, power, and resistance within pandemic responses. We defined community in broadly sociological terms, as ‘a social group circumscribed by geography or experience and bound by a sense of belonging that may be sustained across time and space.’ The multidimensional nature of community indicates ‘shades of meaning’ [30], we thus recognised that interviewees might define or experience community in different ways by focusing on specific dimensions of community [31] or belong to multiple communities (e.g. of knowledge, practice, interest, identity) [32, 33], but chose to focus on community of place (e.g. area-of-control) and of experience (e.g. COVID-19 response) as most appropriate for this research. Recognising how community context plays a key role as a determinant of health outcomes has resulted in advocacy for active community involvement [31, 34]. Interactions of people with each other and within existing community structures provide opportunities for adaptation and improvements in outbreak responses [35].

### Research question

Our research question was intentionally broad, to allow interviewees flexibility in discussing mitigation measures, information sources and local authority responses: “*How have people experienced the COVID-19 pandemic and prevention and mitigation responses by community groups or local authorities in Syria’s three main areas of control*?”

### Sampling and recruitment

Eligibility criteria were: (i) Syrian nationals living in Syria during the COVID-19 pandemic; (ii) aged 18 years or over, able to be interviewed in Arabic or English, and with access to a sufficiently good internet connection to complete a 30–60 minute interview; and (iii) not professionally involved in healthcare or COVID-19 response activities. We purposively ensured at least half our interviewees were women, to help counter Syrian women’s frequent underrepresentation in research [11], and a range of ages and occupations or education levels to provide a diversity of perspectives.

We used two-stage sampling, as recruitment was challenged by safety concerns and internet access. First, we sampled authors’ personal connections (e.g. friends, acquaintances, former colleagues) purposively through WhatsApp, to provide an approximately equal balance of geographic residence and genders along with a mix of ages and occupations/education levels. Second, we snowballed from each interviewee by asking each to identify two potential interviewees. We invited 29 people of whom 7 declined, 4 citing connection problems and 3 citing safety concerns or equating academic research with journalistic exploitation.

### Data collection

We developed a question guide in Arabic (S1 File) from the literature and expert consultation, which we refined iteratively [35–37]. Topics included life changes and biggest concerns since COVID-19, experiences of prevention measures, health information sources, and thoughts about COVID-19 vaccination. We obtained informed consent by sending study information sheets and consent forms via WhatsApp to potential interviewees and arranging individual meetings to discuss questions and concerns. For those choosing to participate, we recorded written (i.e. 17) or verbal (i.e. 5) consent prior to interview, as described by Douedari *et al* [11]. We used a saturation grid, as described by Fusch and Ness [38], to help determine data saturation.

HM, AK, and OA conducted interviews in April-May 2021, at times chosen by interviewees to increase confidentiality, digitally recorded them with interviewee consent (i.e. 2 refused recording), and took extensive notes to contextualise findings. As interviewees were based in Syria, and investigators in the United Kingdom, interviews were conducted using the internet call freeware application WhatsApp (Meta Platforms, US), as it is most familiar to Syrian interviewees and its encryption is suitable for research purposes. Interviews averaged 45 minutes each, were recorded anonymously using numerical identification codes, and transcribed narratively by investigators. We stored password-protected anonymised audio recordings, transcripts, and notes electronically on institutional servers accessible only to investigators. Interviewee identities were anonymised as ‘SU’ (service-user) plus interview number (e.g. SU1 was the first service-user interviewed). An on-call Arabic-speaking psychotherapist was available to provide remote psychological support, though no one made use of this.

### Analysis

MA, HM, and MM analysed data thematically in Arabic and English, using a six-step process informed by Smith *et al*’s interpretive phenomenological approach [39]: (i) reading and re-reading; (ii) initial noting; (iii) developing themes; (iv) searching for connections across themes; (v) moving to the next case; and (vi) looking for patterns across cases. We contextualised themes according to question guide topics and interview notes and translated relevant quotes into English for inclusion. Themes were reviewed by NH and discrepancies resolved between investigators. While location and gender were considered in analysis, it was not possible to analyse consistently on other characteristics (i.e. age, education, occupation). Reporting adheres to COREQ criteria [40].

### Reflexivity

Interviewers, 2 men and 1 woman, were former Syrian health-workers with lived experience of the Syrian socio-political context and master’s level qualitative research training. This background appeared to facilitate trust, interest in participating, and discussion. Investigators’ multi-disciplinary health and social care backgrounds influenced interpretations: MA, HM, MM, and YD have MSc degrees in public and global health, AK is completing a MEd, OA is a medical doctor, YD is a dentist and research fellow, MA and MM are former pharmacists now research assistants, HM is a former pharmacist now freelance researcher, and NH is an associate professor with over ten years’ applied public health research experience in the region.

### Ethics

The Departmental Ethics Review Committee at the Saw Swee Hock School of Public Health in Singapore (reference SSHSPH-093) and Observational Research Ethics Committee at the London School of Hygiene & Tropical Medicine (reference 17360) provided ethics approval, as no legitimate institutional review board existed in Syria at the time of data collection [11].

### Inclusivity in global research

Additional information regarding the ethical, cultural, and scientific considerations specific to inclusivity in global research is included in the S1 Checklist.

## Findings

### Interviewee characteristics and analytical themes

Table 1 provides characteristics for 22 interviewees (i.e. 6 in AACA; 9 in GCA; 7 in OCA). Twelve were women, 3 had postgraduate degrees, 15 had bachelor’s degrees, and 4 had secondary or lower education. Ages were roughly divided, with 2 aged under 20, 5 aged 20–30, 5 aged 30–40, 7 aged 40–50, and 3 aged over 50. We chose interviewees who were laypersons, i.e. not working in healthcare or in the COVID-19 response, and none had specific COVID-19 technical knowledge or experience.

**Table 1:** Interviewee characteristics.

ID	Area	Gender	Approximate age	Education completed	Job
SU13	AACA	Woman	20–30	Bachelor’s	Clerk
SU6	AACA	Woman	30–40	Bachelor’s	Engineering
SU10	AACA	Man	30–40	Bachelor’s	NGO staff
SU11	AACA	Man	40–50	Bachelor’s	Clerk
SU12	AACA	Man	40–50	Bachelor’s	NGO staff
SU5	AACA	Man	40–50	Postgraduate	Academia
SU20	GCA	Woman	18–20	Bachelor’s	Graphic designer
SU16	GCA	Woman	20–30	Bachelor’s	Unemployed
SU14	GCA	Woman	30–40	Bachelor’s	Lawyer
SU17	GCA	Woman	40–50	Bachelor’s	Homemaker
SU15	GCA	Woman	50–60	High-school	Homemaker
SU19	GCA	Woman	61–70	None	Homemaker
SU22	GCA	Man	20–30	Bachelor’s	Lawyer
SU21	GCA	Man	30–40	Bachelor’s	Business
SU18	GCA	Man	61–70	High-school	Retired
SU3	OCA	Woman	20–30	Bachelor’s	Education
SU4	OCA	Woman	20–30	Bachelor’s	Education
SU1	OCA	Woman	18–20	High-school	Media
SU2	OCA	Woman	40–50	Postgraduate	Education
SU7	OCA	Man	40–50	Bachelor’s	NGO staff
SU9	OCA	Man	40–50	Bachelor’s	NGO staff
SU8	OCA	Man	30–40	Postgraduate	Student

We included five inductive themes: (i) reduced fear of COVID-19; (ii) difficulties adhering to NPIs; (iii) unreliable information sources; (iv) limited community-based responses; and (v) vaccination hesitancy. For clarity, we reported thematic findings separately for GCA, AACA, and OCA and used standard terminology (i.e. all, most, half, many, some) as a rough 5-category Likert scale to indicate frequency of topics discussed by interviewees.

### Reduced fear of COVID-19

Most interviewees, across the three areas-of-control, described the fear Syrians experienced at the beginning of the pandemic. However, when the first COVID-19 wave was not as bad as expected, fears about the deteriorating economic situation took precedence and many began ignoring NPIs.

#### Government-controlled areas (GCA)

Most GCA interviewees described overwhelming initial fear and confusion from social media and rumours about the pandemic in other countries, and adhering to prevention measures even before the first Syrian cases were announced.

“*The burden of anxiety on us is more than the burden of the disease itself” SU19*

However, information was confusing and some continued denying the reality of the pandemic.

*“At first*, *we didn’t take the matter seriously […]*. *However*, *there was chaos in health facilities especially public hospitals*…*” SU22*

Many reported cognitive dissonance and confusion as reported deaths increased while media downplayed the pandemic.

*“We started hearing about many deaths*, *which cannot be explained except as COVID-19*!*” SU20*

*“If someone gets infected we wouldn’t know [conclusively] since there are no tests and the diagnosis would be suspected corona*…*” SU21*

All admitted initial fears of severe COVID-19, given the weakened public health system, ongoing economic sanctions, and high costs of private hospitals. They assumed local health facilities lacked capacity to respond.

*“I was so worried because I have asthma and am so aware of the weak status of our health system*…*” SU16*

A major driver for initially adhering to NPIs was worry about family members with chronic diseases.

*“My main concerns were about my father and mother*, *as they are elderly and very vulnerable*. *Even at Eid* [Muslim holiday with communal gatherings], *we did not gather as we were so afraidi*…*” SU17*

As young men left Syria, many interviewees described elderly people having to increasingly support families financially, increasing their COVID-19 infection risk.

*“Most of these people are elderly*, *as there are fewer young people in the city because of the events* [conflict]. *That means if they [*elderly*] decide to take the risk and go out*, *they are more likely to get seriously ill*. *So sad to see those elderly people risking their lives like this*, *where they are at an age where they are supposed to get all the services*, *health and social care*, *to protect them from any harm” SU20*

Some discussed the mental toll of the pandemic, including developing obsessive hygiene practices.

*“It has made us very anxious—lockdown*, *obsessive cleaning*, *anxiety if we have touched anything*, *any kid goes out and gets back we make them shower—this has all been exhausting” SU19*

Many described how stigma against those who were infected or had to self-isolate negatively affected their wellbeing.

*“The mental health effects of [COVID-19]*, *for people who are most vulnerable or who have got it*, *the stigma and self-isolation all affect people” SU17*

However, they also described a form of instinctive resilience.

*“We in the country were less stressed or anxious [than overseas relatives]*. *I guess it’s just because we need to be like that*, *otherwise we would have serious mental health problems” SU20*

Most GCA interviewees described their shift from “overreacting”, as they saw people getting mild COVID-19 symptoms and not dying, and their fears reduced.

*“Eventually*, *we got back to normal life*. *Of course*, *personal hygiene is always in place*, *but we do not use disinfectants anymore*, *because we are not afraid anymore*. *We got it [COVID-19] and it’s not as scary as we thought*.*” SU16*

#### Autonomous administration-controlled areas (AACA)

All AACA interviewees similarly described initial fears about this dangerous virus reported in international media, given health system weaknesses and not yet having met anyone infected or recovered from SARS-CoV-2.

“*In Europe and the US*, *with all the developed health systems and resources*, *they faced a lot of problems and they were not able to contain the disease*. *So*, *what would happen to us*?*”* SU10

However, many interviewees described how worries faded as numbers of locally-reported cases, particularly severe cases, were relatively low.

*“The small number of cases in the area reduced people’s fear*, *as these cases were mild and healed quickly*.*” SU6*

#### Opposition-controlled areas (OCA)

Most OCA interviewees described similar fears when initial COVID-19 cases were announced, primarily linked to limited health system resources and information.

*“COVID-19 wave*, *coupled with weak health infrastructure affected us a lot in the first days after announcement of the first case*. *People were scared and there was a state of chaos all around*, *because there wasn’t much public information available about COVID-19” SU7*

As reported case numbers remained low, compared to neighbouring countries, they described how people became less afraid and less willing to adhere to NPIs given ongoing economic hardships.

*“Later on*, *as the number of recovered cases increased*, *and not many deaths were recorded*, *the fear and anxiety of getting infected with COVID-19 decreased*. *I think most of us got somehow infected by COVID-19 including myself*, *but I didn’t take a PCR test” SU1*

Following declining case numbers after the first COVID-19 peak in November 2020, all interviewees described few prevention measures being followed.

*“People forgot that there ever was a pandemic and life is back to normal*.*” SU3*

### Difficulties adhering to NPIs

Most interviewees considered many recommendations and interventions to be disconnected from essential community needs for food and income security. For example, providing masks for people living in small family tents and sharing toilets, or asking daily-wage earners to self-isolate without compensation, simply reduced community trust and goodwill towards local health authorities. Reasons suggested for the difficulties adhering to NPIs only differed somewhat across areas-of-control, as described below.

#### GCA

*Religion*. Some reflected how Islam prioritised disease prevention.

*“What helped us to adhere to lockdown measures is our belief in Islam*. *Prophet Mohammed (PBUH) was asked [what to do] when an outbreak happened*. *He told people to stay home” SU19*

One mentioned how faith did not require her to accept misinformation, such as Muslims being protected from COVID-19 because of ‘wudu’ (ritual ablutions before praying). Some suggested perceived fatalism misinterpreted religious teaching.

*“Some people say that God is the protector*. *Yes he is*, *but we need to do all we can to prevent it [COVID-19]*!*” SU17*

*Socio-cultural norms*. Most interviewees described safe distancing as hardest given required service systems, such as queuing for bread every morning, staying home due to evening curfews, and navigating crowded transportation. Any outside interaction meant not complying with safe distancing. Working from home was not possible for many Syrians because of poor internet and irregular electricity. Many complained about expensive facemasks and none mentioned effective reusable masks. None described any community engagement by local authorities to help address concerns and all noted the impracticality of NPIs for most Syrians, especially for poorer people.

*“Preventive measures do not make sense to me anymore*. *I mean*, *I have to go to the university using public transport*, *which is very crowded*, *and I have to use it because I cannot afford private transport*. *On the bus*, *no one wears a facemask*, *and you cannot keep a proper distance*. *So*, *I feel what’s the point for me to use a facemask or mind social distance*?*” SU16*

Additionally, some who tried to implement NPIs reported experiencing social isolation.

*“I felt very strange wearing facemasks and being the only one*!” *SU18*

*School closures*. Many interviewees described how education shifted online without any engagement with students and parents on necessary contextual adjustments. Students were required to attend year-end exams, even with COVID-19 symptoms, to avoid failing. Educational institutions had no unified policies, with lengths of closures and flexibility for students varying across GCA. Logistical challenges were similarly unaddressed.

*“As universities stopped physical teaching*, *they intended to move to online education*, *but this did not happen*, *simply because the university does not have the resources to make the lectures virtual and people do not have the tools*, *electricity and internet*, *so basically*, *we lost the academic year*. *We have not got any education*, *yet we’re supposed to attend tests and pass exams*!*” SU20*

*Livelihoods*. All GCA interviewees described the worsening economy as the main challenge to NPI adherence. Many were already struggling to provide for families, and pandemic responses such as lockdown amplified these struggles through increased commodity prices, reduced remittances—on which many Syrians depended, and inability to work.

*“All people are under financial pressures*, *and no one is able to help others” SU21*

Some indicated solidarity for those even less fortunate in speaking about the lack of public engagement by local authorities when imposing COVID-19 restrictions in a context in which many people needed to leave home each day earn money and buy supplies to survive.

*“They cannot afford to feed their children and the advice is to stay home*. *How could they*…?*” SU15*

These struggles were exacerbated by limited affordable medications and reliance on private healthcare because of lack of trust in public facilities. For example, someone with suspected COVID-19 might avoid public facilities for fear of definitely contracting COVID-19 there and spending more than a month’s salary for treatment. No interviewees described any community engagement or communications efforts by health authorities to help alleviate these concerns.

*Uncontextualized awareness campaigns*. Many interviewees noted lack of awareness as an obstacle to adherence, worsened by disconnections between reality and campaign materials due to the general lack of public consultation or engagement by local authorities. They suggested that the lack of updated COVID-19 information left people thinking COVID-19 was less relevant than in other countries.

*“TV coverage [of COVID-19] has decreased*. *They used to talk about it a lot*. *Now it is less and they do not pay much attention to it” SU15*

*Weak health system governance*. All reported public health responses as chaotic and confusing, with no transparency about numbers of COVID-19 cases and deaths. No support was provided to the public or health providers, with most patients having to buy their own medical equipment and oxygen to use at home. Government health facilities lacked employee sick-leave policies for COVID-19. Many health providers reportedly did not comply with prevention measures and PPE, increasing cases and deaths among providers and reducing public concerns about COVID-19, as health providers were considered role models.

*“All the institutions do not have policies for regular infection control*. *Even at hospitals*, *doctors do not always wear facemasks*. *I mean*, *a patient—who is less educated about health—will then think facemasks are not very important*. *My point is*, *we need policies at a very high level so people can adhere*…*” SU20*

No interviewees mentioned the government’s role in pandemic response, unless explicitly asked, suggesting government was not active in the COVID-19 response.

*“The government is not able to tolerate the burden on its own and the patient also is not able to hold this burden*” *SU22*

Interviewees indicated the government was not responsible for providing facemasks, PCR tests, PPE, or financial support, nor did it regulate prices or availability of drugs and equipment. All interviewees, when asked explicitly about government contributions, described its response as providing advice but no regulations or enforcement, except for evening curfews managed by security forces.

*“The advised measures were different from the implemented ones*. *There was no mechanism to impose measures” SU22*

Many interviewees described exploitation of pandemic responses and possible corruption. For example, security forces reportedly sold pass cards during curfew, managed (for a cost) funeral requirements for people who died of COVID in public hospitals, subcontracted children to sell expensive facemasks at entrances of government offices and required people to mask inside, and imposed monopolies on disinfectants and facemasks sold on at inflated prices.

*“There was no regulation from government […]*. *Things were chaotic and corrupt” SU21*

#### AACA

*Religion*. The perception that everything was in God’s hands was particularly prevalent in AACA, with most interviewees discussing religion.

*“The religious factor [belief in fate] made some people ignorant about getting infected*, *its seriousness*, *and the possibility of death” SU6*

*Socio-cultural norms*. Traditions vital to social wellbeing, such as shaking hands or kissing when greeting, challenged infection control measures.

*“We are a tribal society*. *We have social commitments*.*” SU10*

*School closures*. Most interviewees were particularly concerned about educational interruptions, describing mitigations through remote education, as *“shy and limited” (SU10)* due to insufficient planning and resources. Some suggested that halting education in AACA was worse than in other areas due to previous lengthy conflict-imposed interruptions.

*“Schools reopened in the area just two years ago*, *so it is a very critical time for the students to build motivation and enthusiasm about it… Online education is not working for us at all*. *It destroyed what we’ve been building*.*” SU10*

*Poverty and empty options*. The pandemic halted livelihoods and education for most. All interviewees expressed more concern about losing income than COVID-19 infection. Adherence to safe distancing and other NPIs was considered a luxury.

*“People are scared they might get infected*, *but they just cannot afford not going to work*.*” SU12*

Livelihoods were negatively affected by both pandemic and NPIs. Type of occupation determined adherence, with many interviewees explaining that people working for international organisations more able to adhere than those in precarious jobs or who could not work from home.

*“The simple worker who needs food*… *you have no power over him since you are not responsible for securing his basic needs*!*” SU6*

*Weak health system/coordination*. All interviewees described AACA health system performance as very limited, with weak health authorities, underdeveloped services, and severely lacking medical equipment and supplies due to ongoing conflict, financial constraints, and reliance on inconsistent international aid.

*“The health situation in our area is very bad*. *Medicines are expensive […]*. *There are only two public hospitals*, *and the rest are private […]*. *They have been waiting for promises of more aid*, *but nothing happened” SU6*

Major challenges to NPI implementation, mentioned by all interviewees, were lack of cohesion of rules and responses between health and political decision-makers and lack of community engagement.

“[AACA authorities] *should never interfere in medical issues… setting the pandemic rules and measures was so incorrect and led to huge problems*. *These are purely medical matters and politicians should have no say in them” SU5*

Most interviewees criticised some COVID-19 responses, particularly curfew and partial lockdown, as ineffective or even worsening crowding and decided without any public consultation.

“*It has a huge bad effect on the numbers of cases as it led to more people gathering outside the lockdown window” SU5*

However, a dissenting suggestion to increase NPI effectiveness was through legal consequences for non-adherence.

*“Health facilities’ role was limited to awareness*. *I think fines or other penalties should be the way to enforce the movement ban and lockdown measures” SU13*

#### OCA

*Religion*. Religion had contradictory effects, with many OCA interviewees describing the positive role of ritual cleanliness related to handwashing and avoiding infection, while some assumed religion would have to protect them irrespective of prevention measures.

*“At the beginning we used to wash our hands and avoid shaking hands when meeting other people*, *but not anymore*. *God is our only protection” SU3*

*Socio-cultural norms*. Most interviewees indicated norms, such as visiting relatives, were primarily responsible for Syrians not adhering to safe distancing despite increased risk to elderly relatives.

*“Our social norms are very important in our culture*, *such as visiting our mother*, *relatives*, *and close family members*. *Even though you fear for their health*, *norms and cultural habits push you to break safe distancing*.*” SU9*

*Poverty and lack of options*. All interviewees suggested income insecurity was the main barrier to prevention measures such as work-from-home and self-isolation. Prevention was an “*empty option*” (SU7) for daily-wage earners for whom missing a day meant their family did not eat. Thus, people risked COVID-19 rather than family starvation.

*“People didn’t have options; the majority of people are obliged to work*. *They either get infected with COVID-19 or they and their children die from hunger*. *So*, *people chose to continue working*. *They said if we get infected then it is our fate” SU9*

Among displaced communities, camp crowding and poverty hindered adherence.

*“People here are suffering from poverty*, *unemployment and displacement*. *How can you ask those living in a tent to self-isolate*?*” SU2*

Even for those who could afford to self-isolate, all interviewees described the lack of infrastructure for meeting everyday needs without leaving home, forcing people to visit markets for daily household supplies.

*“At the end you are obliged to get out of the house to work and to bring groceries to your family*. *So life here obliges you to go to local markets for shopping*.*” SU9*

*School closures*. One interviewee praised the relatively rapid transition to online education during COVID-19 lockdown, as schools had prior experience due to conflict and school attacks and reverted rapidly online.

*“In the first few weeks after the announcement of COVID-19*, *there was lots of confusion*. *However*, *we have prior experience in remote teaching due to the conflict*. *So*, *schools were fast in moving children into online education*. *As a result children didn’t miss more than two weeks of education*.*” SU7*

However, most highlighted that the quality of their children’s education deteriorated. Students attending WhatsApp classrooms suffered internet disruptions due to electricity shortages, while many parents did not have smart phones for WhatsApp group calls.

*“Education was interrupted by school closures and they tried continuing online education*, *but this is complicated*. *Many people don’t have access to internet and online communication*, *including access to electricity*. *Therefore*, *education outcomes decreased and many students don’t have computers or devices to help them continue their education online*.*” SU8*

*Poor response coordination*. Most interviewees suggested insufficient coordination among health and COVID-19 response actors was the main hindrance to rigorous COVID-19 NPIs.

*“The Ministry of Health and Health Directorate may have administrative roles only*. *They lack power to impose restrictions*. *Organizations have a big role on the ground*, *yet each has its own separate projects and regulations” SU2*

Despite significant investments in establishing COVID-19 community isolation centres in OCA, with one interviewee mentioning a centre in each village, most interviewees were afraid to stay in them.

*“There were many community isolation centres*, *yet they were badly equipped*, *very cold*. *If we don’t die from corona we would die from cold*. *Many of these centres were just tents*, *not a proper building*.*” SU3*

### Unreliable COVID-19 information sources

Most interviewees considered local health information sources unreliable, except for COVID-19 statistics in OCA. Social media and WHO websites were considered better.

#### GCA

Most interviewees described international media outlets as more trustworthy than GCA ones. Common information sources reported were WHO for reliability, social media—especially Facebook—for accessibility, and local health professionals/specialist acquaintances for advice. Despite distrust in, and lack of transparency of, local news sources, most interviewees suggested local doctors were objective and realistic. Many interviewees suggested that as information was repeated it became more believable, though some indicated they would check new information with their doctors or pharmacists. One called the international news on COVID-19 terrifying and suggested media outlets were exaggerating. All but one indicated social media as primary information source, though most noted that not everything on social media was reliable.

*“Of course*, *not everything on the internet is correct*, *you need to use your common sense*.*” SU19*

Most interviewees wanted information on how to differentiate between COVID-19 and other respiratory infections, since PCR testing was unavailable, maximum virus infectiousness on surfaces, justification of high-risk groups, the impact and future of the pandemic, and exit plans including vaccine safety and effectiveness. One suggested using mosques as campaign centres might increase impact. Another suggested simplified messages focused on the elderly, since younger people were at less risk.

#### AACA

All identified information sources as primarily social media, particularly Facebook, WhatsApp, and YouTube. Some mentioned following social media channels of doctors in OCA and abroad to determine credibility, but only one questioned the veracity of YouTube and Facebook as information sources and seemed aware of misinformation. Anecdotal experiences from relatives, friends, and colleagues were an important information source for all interviewees, while official news outlets were regarded with suspicion due to the lack of real-time statistics on COVID-19 cases and deaths. Most interviewees wanted more information on the future of the COVID pandemic and vaccine effectiveness and safety.

#### OCA

All interviewees similarly described social media as their main COVID-19 information source. Despite most reporting information from Idlib health directorate as credible, it was not sufficient. Most also identified WHO information as evidence-based and trustworthy.

*“In my opinion*, *WHO is the most credible channel for COVID-19 information*, *and disseminating related information*. *Social media is our source of information […]*. *I don’t follow the local channels and news*. *They aren’t a reference for me or in general for people here” SU8*

Views about the role of religious leaders in sharing COVID-19 information were contradictory, with one suggesting that many religious leaders were sharing misinformation, while another suggested organisations could use religious leaders for dissemination as they influenced most of the OCA population.

*“Even though [Idlib] Health Directorate was disseminating information about COVID-19*, *was this information reaching the public*? *I don’t think so*. *It was mainly directed toward the educated population*. *Thus*, *we lack the community awareness that could be reached through the mosques and Friday prayers […]*. *Our religious leaders themselves lack awareness about COVID-19*.*” SU7*

### Limited community-based responses

People in most areas relied on individual or household prevention rather than organising community initiatives, reiterating that COVID-19 response engagement with/by local authorities was very minimal.

#### GCA

Most interviewees described behavioural changes, physical distancing, not shaking hands, and disinfecting everything, with households creating new hygiene habits for COVID-19.

*“The measures followed were personal more than societal*.*” SU21*

Some focused on nutrition and attempting to boost their immune system.

*“I have a belief that if people are taking care of their health*, *eating well and taking simple measures*, *they won’t get ill*.*” SU17*

There was minimal consistency among interviewees—depending on disease knowledge and risk perception—and a focus on protecting their household rather than collective responsibility for protecting the community or society.

*“I mean at the beginning it was like an OCD*, *and this started to fade*, *maybe after the first six months*. *Now we are committed to more realistic measures like face-masks and regular hand disinfection*.*” SU20*

Some interviewees suggested improving COVID-19 responses through providing food to households prior to imposing lockdown—as livelihoods depended on daily earnings, supporting the health system and facilities, and providing cash for people to buy facemasks and disinfectants.

*“Awareness is not the problem*. *The main need is in health system capacity*! *Stretched staff and weakened health services*. *Here we should do more initiatives; more funds from international aid to support the health system or cash transfers for daily wagers who have been affected by the lockdown” SU16*

Community initiatives varied, with some interviewees saying they did not know of any, while some mentioned: (i) shop owners trying to control commodity purchasing and stocking to reduce prices; (ii) pharmacies reducing medicine prices for COVID-19 patients; (iv) doctors offering telemedicine consultations to reduce pressure on health facilities; and (v) communities collecting unused medications and initiating a medication bank at pharmacies to mitigate sharp price increases. Some interviewees identified NGO initiatives as community-based, indicating these were insufficient given the need.

*“There were campaigns to distribute facemasks etc […]*. *All of these are luxuries compared to food items*. *There are people who cannot afford to buy bread*. *[…] When someone feels full*, *they can care about Corona*. *If you provide facemasks and disinfectants to any household*, *they will not be grateful because these are not their priorities” SU21*

*“At the beginning of the pandemic here*, *a charity implemented a campaign where they distributed flyers*, *which I think was bizarre*! *It can help spread the virus*!*” SU14*

#### AACA

All interviewee accounts agreed that community initiatives were limited, primarily hindered by lack of awareness, competing priorities, and limited funding. Interviewees mentioned local initiatives, including making reusable fabric masks, *“but they stopped due to lack of support*, *which is so expected” (SU12)*.

Most interviewees noted that higher-level efforts, particularly increasing health system resources, would improve the response. Ideas included separating COVID-19 treatment centres from hospitals, increasing laboratory numbers, and correcting misinformation.

*“We need a single specialized entity to publish reliable information*…*” SU11*

#### OCA

Some interviewees suggested the importance of sharing personal experiences to strengthen community trust and awareness.

*“After I got infected with COVID-19*, *I developed my own experiences of the disease*, *symptoms*, *and how to recover*. *And I started passing this information to others*. *We were sharing among ourselves information and experiences of COVID-19*, *how I was infected*, *how I recovered” SU8*

One suggested theatre performances, while emphasising the contextualisation of any initiatives.

*“I would suggest short theatre plays for mothers and children to educate them about the importance of not shaking hands and kissing […]*. *Community messages should vary according to different social groups*.*” SU2*

Almost all agreed that Syrians needed financial support more than awareness messages.

*“An idea without funding will fail*. *Though awareness campaigns are important*, *people need support and money*.*” SU3*

### COVID-19 vaccine hesitancy

Most interviewees expressed hesitancy about COVID-19 vaccination, related particularly to insufficient information or trust in its origins and local cold-chain.

#### GCA

Perceptions of COVID-19 vaccination varied. One mentioned that differences among vaccine brands and countries of origin would affect his decision. Another suggested that national health centres were trustworthy places to administer vaccines. Two stated they would not be vaccinated or allow their relatives to get it, given its short development period was insufficient to ensure safety. One voiced support for conspiracy theories.

*“I don’t trust it [the vaccine…]*. *If I were for some reason forced to take it*, *I would*, *but if it is optional I wouldn’t*.*” SU22*

#### AACA

Only one interviewee expressed willingness to be vaccinated if offered, while others were hesitant for either themselves or their relatives to be vaccinated, especially if they could not verify vaccine source (i.e. brand and country of origin) and efficacy or hear positive stories from those already vaccinated.

#### OCA

No interviewees expressed interest in being vaccinated, mainly because they were convinced they had been infected with COVID-19 and were relatively young and therefore did not need it.

*“I wouldn’t take it*, *because the vaccine is still under development*. *And myself I got infected with COVID-19*. *I only felt like severe cold symptoms and then I recovered*, *but I didn’t feel it was very dangerous for me*. *So*, *I think it may be useful for a small group of people but not for me*.*” SU8*

One interviewee highlighted the negative impact of rumours, particularly that COVID-19 vaccine is developed by the Russians to kill the remaining OCA population.

*“In general people aren’t excited about the vaccine*. *Rumours say that Russia will send the vaccine to kill us all” SU2*

Another suggested that if health-workers shared their positive vaccination experiences, people would be less hesitant.

*“If we can hear from health staff who received the vaccine about their experiences*, *and from the health sector about the vaccination process and from where it was imported*, *then we would feel more confident about taking the vaccine” SU1*

## Discussion

This study is a first effort to explore perspectives and experiences of COVID-19 responses among ordinary Syrians in three areas of control in Syria. We highlighted similarities and differences in a country divided by a protracted ten-year conflict. Our approach contributes to decolonising global health research, in being led by former Syrian health-workers with insider knowledge of the Syrian conflict and health systems [11].

Across our five themes of reduced fear of COVID-19, difficulties adhering to NPIs, unreliable information sources, limited community-based responses, and vaccination hesitancy, we found that the main issues raised were the primacy of fearing income loss over COVID-19 infection, lack of effective community engagement by local authorities and thus inappropriateness of many standard NPIs, focus on personal/household-level prevention rather than local authority efforts, trust in God whether as motivation to increase or reduce prevention efforts, and substantial vaccine hesitancy due to unreliable information, distrust, and rumours.

The fear of income loss appeared understandable given the precarity of many people’s lives in Syria and the challenges caused by NPIs that threatened education, livelihoods, and survival. In addition to increased local constraints, the global COVID-19 impact reduced the remittances that are the main support for many Syrians. Braam *et al* describe this also occurring in Somalia, which similarly relies heavily on remittances [41]. COVID-19 interventions must consider socioeconomic realities. For example, Loewenson *et al* showed that community initiatives to provide food and financial support for families during quarantine increased adherence [42]. Local authorities must consider the long-term cost-effectiveness of NPIs such as lockdowns and school closures in low-income conflict-affected settings such as Syria [43].

Related to our findings of fear of income loss outweighing concerns about COVID-19, lack of community engagement was particularly highlighted in GCA and to a lesser extent AACA, while in OCA interviewees focused more on the lack of coordination and consistency between multiple authorities and actors. Gilmore *et al* highlighted the importance of community engagement to frame equity-informed COVID-19 responses, as communities can play active roles in disease control if interventions are applicable within their contexts [44, 45]. Responses to HIV and Ebola similarly demonstrated the effectiveness of community engagement [2]. However, our findings indicated community engagement was poor across Syria with many COVID-19 response interventions disconnected from community needs. For example, requiring lockdowns, school closures, and self-isolation in a socioeconomically precarious context without work-from-home options and with authorities unable to compensate people [15, 41], can only increase people’s lack of trust in local authorities. Engagement exists on a progressive spectrum from the minimum of informing, through consulting, involving, collaborating, to empowering [45]. However, community engagement by authorities in COVID-19 responses across Syria was insufficient even for the minimum of informing. Factors to consider in improving community engagement in Syria include: (i) avoiding "one-size-fits-all” approaches and ensuring the needs of the most marginalised are included; (ii) improving transparency and responsiveness so people’s concerns can be addressed or at least considered; and (iii) accommodating grassroots initiatives such as "*sterilize it”* [46] in Damascus and engaging beyond the pandemic [2].

Fears of income loss, incompatibility of many imposed NPIs with people’s lived realities, and insufficient attempts by local authorities to engage with communities, combined with fragmented and poorly coordinated health systems in the three areas-of-control, meant most people appeared to rely on personal/household-level prevention and religious faith. However, personal prevention and mitigation require sufficient accurate information to make informed choices and many Syrians relied on whatever the internet provided (on different social media platforms and according to their algorithms). Access to information is a basic right that authorities should facilitate, with Human Rights Watch emphasising “*Governments are responsible for providing information necessary for the protection and promotion of rights*, *including the right to health*” [47]. The COVID-19 pandemic has demonstrated both the importance and violation of this right by authorities globally [48, 49]. Transparency from authorities about cases, deaths, recovered numbers, progress on vaccine development, and growing understanding of the disease reduces anxiety and confusion [50], even in conflict-affected settings such as Syria. It can also reduce the power of misinformation and vaccine hesitancy [51]. More efforts are needed to reach Syrians with accurate health messages using appropriate and accessible social media platforms. Investing in coproduction of communication campaigns [2] and leveraging trust in religious leaders could help engage communities.

Faith was a powerful motivator and justification. Including religion as a health determinant and involving faith communities can be especially relevant during pandemics [52]. We found that Islam was used to both encourage and discourage prevention efforts, emphasising the value of integrated communication approaches that include religious leaders in social mobilisation and correcting misinformation about COVID-19 [2, 41].

WHO reports Syria as among the countries with the lowest COVID-19 vaccination coverage worldwide, with less than 5.3% of the population vaccinated by January 2022 [53]. Our findings show most interviewees expressed vaccine hesitancy, whether for safety reasons or fears of inadequate production source or cold-chain management. Similarly, an online questionnaire of 3,402 participants in Syria, showed 66% of adults rejected COVID-19 vaccination for fear of side effects, while 41% doubted its efficacy [54]. Additionally, misinformation has exacerbated confusion and anxiety, encouraged by people’s lack of trust in local authorities and media [51]. Distribution of COVID-19 vaccines will be challenging in Syria, due to years of mistrust in authorities and fragmented health governance. COVAX partners must consider appropriate and meaningful community engagement that accounts for these ongoing issues if the roots of vaccine hesitancy in Syria are to be addressed.

Several limitations should be considered. We aimed for a snapshot of community experiences across Syria as this is a small exploratory study. Conceptual transferability of findings should be done carefully as sub-regional histories and experiences differ. While all interviewers interviewed women, only male interviewers interviewed men. However, differences were not detected in women’s responses whether they were interviewed by a man or woman. OA and AK were relatively new to remote qualitative interviewing, so some nuances may have been missed. Finally, we aimed for data saturation rather than attempting to obtain a large enough sample to examine differences in age, education, occupation, or ethnicity and future research could address this.

## Conclusion

COVID-19 responses across Syria have not sufficiently engaged or considered communities. NPIs did not address people’s fears, consider basic socioeconomic needs, or actively seek to improve trust or correct misinformation. Thus, many response interventions were ignored or rejected as the pandemic progressed. Meaningful community engagement, in which community members are considered as partners or leaders and engagement is a two-way learning process, is crucial for COVID-19 mitigation and future infectious disease responses, to improve adherence to public health measures, correct misinformation, and address vaccine hesitancy.

## Supporting information

S1 File Interview question guide (Arabic and English translations). (PDF)

S1 Checklist Inclusivity in global research checklist. (PDF)
