# Supplementary material for: “They cannot afford to feed their children and the advice is to stay home. How‥?”: A qualitative study of community experiences of COVID-19 response efforts across Syria
Source: PLoS One. 2022 Nov 4;17(11):e0277215. doi: 10.1371/journal.pone.0277215 (PMC9635699; doi:10.1371/journal.pone.0277215)
Supplement: S1 File — (PDF) [file pone.0277215.s001.pdf]

Alhaffar *et al.* Interview question guide (English)

INTERVIEWER NOTE: Please record ID code at the beginning of the recording and on your notes. Do not say interviewee’s name. Remember, questions are indicative only, as some topics will be more interesting for participants and some topics may be irrelevant for them.

READ: *Please confirm that you have been informed about this study, your questions have been answered, you understand that if you wish to avoid a question or stop at any point you may do so, and that you are participating willingly.

1. How has life changed for your family since COVID-19 began? [Explore]
    a. How has COVID-19 pandemic affected your area and what are your biggest concerns, e.g. livelihoods, education, or other issues?
    b. On a scale of 1-10 (10 being worst) how concerned are you, or someone in your family, getting COVID-19 (and why)?

2. What COVID-19 prevention measures are being implemented or advised in your area and how has adherence changed during the pandemic?
    a. Are you and your household able to adhere to these prevention measures (why/why not)?
    b. Are you aware of any support provided by health actors (e.g. distribution of PPE or soap)?

3. What practical things do you think could be done to mitigate the effects of COVID-19 on people in your area? [Explore, e.g. giving masks, supporting handwashing, enforcing safe distancing, providing isolation/treatment facilities]
    a. Are there any community-led responses to COVID-19 in your area that you could describe? [Explore: How effective/successful do you think they are? What could be done to strengthen them?]

4. How do you get information about COVID-19? [Explore: e.g. which people/institutions, how trustworthy is it, and what are they advising?
    a. What kind of information would you like to receive on COVID-19 and what would be the best way for you to access it?

5. If you were offered COVID-19 vaccination, would you take it (why/why not)?

Thank you for your responses. Is there anything you think we missed or that you would like to talk about?

END

Alhaffar *et al.* Interview question guide (Arabic)

ملاحظات الباحث: سجّل الرقم التعريفي في بداية التسجيل وفي ملاحظاتك. لا تذكر اسم الشخص الذي تتم مقابلته. تذكر أن الأسئلة توجيهية فقط ، حيث أن بعض المواضيع قد تكون مثيرة لاهتمام بعض المشاركين و تتطلب بعض النقاش و بعض المواضيع قد تكون غير ملائمة للبعض الآخر و يمكن تخطيها.

*اقرأ التالي: الرجاء التأكيد أنّه تم إخبارك بهذه الدراسة و تمت الإجابة عن أسئلتك و أنك على علم بأن لديك كامل الحرية بعدم الإجابة على سؤال لا ترغب/ين بالإجابة عليه و لديك الحرية بالتوقف متى تريد/ين و أنك موافق/ة على المشاركة طواعيةً"

1. **كيف تغيرت الحياة بالنسبة لعائلتك منذ بدأت جائحة الكورونا؟** (اسبر)
   أ. كيف أثرت الجائحة على منطقتك و ما أكثر شيء يثير قلقك (مثلاً تأمين المعيشة ، التعليم، أو أي أمور أخرى)؟
   ب. من 1-10 (باعتبار 10 هو الأسوأ) كيف تقيّم قلقك أنت أو أحد أفراد عائلتك من الإصابة بالكورونا؟

2. **ما هي الإجراءات الوقائية من الكورونا التي يتم تطبيقها أو النصح بها في منطقتك؟ و كيف تغير الالتزام بها خلال فترة الجائحة؟**
   أ. هل أنت أو عائلتك قادرون على الالتزام بهذه الإجراءات (لماذا/لماذا لا؟)؟
   ب. هل لديك أي معلومات عن أي دعم مقدم من الجهات الصحية الفاعلة (مثلا توزيع معدّات الوقاية الشخصية أو الصابون)؟

3. **ما هي برأيك الأمور العملية التي يمكن عملها لتخفيف أثر الكورونا على الناس في منطقتك؟** (اسبر: توزيع الكمامات، تعزيز غسل اليدين، تطبيق التباعد المكاني، تأمين مراكز عزل أو علاج)
   أ. هل هناك مبادرات مجتمعية للتعامل مع الكورونا في منطقتك؟ (اسبر: كيف تقيم فعاليتها أو نجاحها؟ ما الذي يمكن عمله لتقويتها؟)

4. **من أين تحصل على معلوماتك المتعلقة بالكورونا؟** (اسبر: ما مدى موثوقية هذه المعلومات برأيك؟ ما هو مصدر المعلومات بالتحديد أشخاصاً أو مؤسسات؟ ما الذي ينصحون به؟)
   أ. ما هو نوع المعلومات المتعلقة بالكورونا التي ترغب بالحصول عليها و ما هي أفضل الطرق للوصول لهذه المعلومات بالنسبة لك؟

5. **إذا عرض عليك تلقي لقاح الكورونا، هل ستأخذه؟ (لماذا/لماذا لا؟)**

شكراً لك . قد وصلنا لنهاية الأسئلة ، هل هناك أيّ شيء آخر ترغب أن نناقشه أو نضيء عليه؟

النهاية
